# Food Derived Bioactive Peptides and Intestinal Barrier Function

**DOI:** 10.3390/ijms151222857

**Published:** 2014-12-09

**Authors:** Olga Martínez-Augustin, Belén Rivero-Gutiérrez, Cristina Mascaraque, Fermín Sánchez de Medina

**Affiliations:** 1 Department of Biochemistry and Molecular Biology 2, CIBERehd, University of Granada, Instituto de Investigación Biosanitaria ibs, Granada 18071, Spain; 2 Department of Pharmacology, CIBERehd, University of Granada, Instituto de Investigación Biosanitaria ibs, Granada 18071, Spain; E-Mails: belenxi@correo.ugr.es (B.R.-G.); fsanchez@ugr.es (F.S.M.); 3 IBD Center, Laboratory of Immunology in Gastroenterology, Humanitas Clinical and Research Center, Milan 20089, Italy; E-Mail: cristimascaraque@gmail.com

**Keywords:** bioactive peptides, food proteins, functional foods, nutraceuticals, intestinal barrier function, mucus, immunoglobin A (IgA), innate immune response, inflammation

## Abstract

A wide range of food-derived bioactive peptides have been shown to exert health-promoting actions and are therefore considered functional foods or nutraceuticals. Some of these actions are related to the maintenance, reinforcement or repairment of the intestinal barrier function (IBF) whose role is to selectively allow the absorption of water, nutrients and ions while preventing the influx of microorganisms from the intestinal lumen. Alterations in the IBF have been related to many disorders, such as inflammatory bowel disease or metabolic syndrome. Components of IBF are the intestinal epithelium, the mucus layer, secretory immunoglobulin A and cells of the innate and adaptive immune systems. Here we review the effects of food derived bioactive peptides on these IBF components. *In vitro* and *in vivo* effects, both in healthy and disease states, have been reviewed. Although limited, the available information indicates a potential for food-derived peptides to modify IBF and to contribute to disease treatment, but further research is needed to better isolate responsible peptides, and to help define their mode of action.

## 1. Introduction

Dietary proteins feature peptide sequences in their structure that are released as actual peptides by natural or artificial proteolysis and which may become physiologically active in the process. Processes that lead to bioactive peptide release include *in vivo* enzymatic digestion in the gastrointestinal tract both by human and microbiota enzymes, and *in vitro* food processing or ripening by starter cultures of microorganisms or by enzymes from animals, plants or microorganisms [1]. A range of health-promoting properties have been attributed to these bioactive peptides, including antihypertensive, anti-microbial, anti-oxidative, immune-modulatory, opioid and mineral binding properties [2,3,4]. Any protein source can originate bioactive peptides, milk being the best studied for obvious reasons, but bioactive peptides from egg, fish, meat, algae or soy have also been reported [2,5,6,7,8].

**Figure 1:**
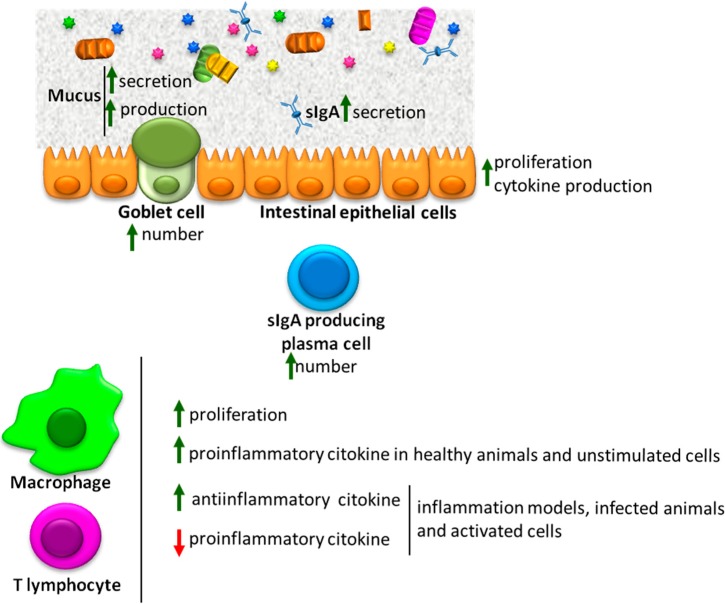
Overall effects of bioactive peptides on several components of the intestinal barrier function. ↑: enhanced; ↓: inhibited.

This review updates the reported effects of dietary bioactive peptides on intestinal barrier function (IBF) (Figure 1). Studies dealing with the effect on the microbiota have not been included.

## 2. Postbiotics

Because fermentation by lactic acid bacteria and yeast results in hydrolysis of milk proteins, yoghurt, kefir and other fermentation products also contain bioactive peptides [1,4,9,10]. As a rule the studies carried out with fermented milk proteins include, as a result of the process of production, compounds such as exopolysaccharide or bacteriocins, and sometimes parts of bacteria or dead bacteria. Thus caution must be taken when interpreting the data, even though peptides are likely to account for the reported effect.

Products obtained after bacterial fermentation in which bacteria have been removed or killed are frequently termed postbiotics [11]. Studies have found that the administration of fermentation products containing live bacteria may be more beneficial than postbiotics. This is the case of studies in which these products were used to treat malnourished animals [12,13]. Nevertheless, the administration of postbiotics could be better and safer than the probiotic or fermentation products containing living bacteria in cases where the bacteria can induce reactions such as acute inflammation [11,14]. The best examples include the study in which patients with acute pancreatitis experienced increased mortality after administration of a combination of three probiotics [15] and the study with an organ culture system of human healthy and IBD intestinal mucosa, where probiotics induced tissue destruction [16].

## 3. Intestinal Barrier Function

The intestine has the essential function of absorbing water and nutrients for the support of bodily functions, thus contributing also to ionic homeostasis. At the same time, it has to keep at bay a rather large amount of microorganisms (and microbial molecules) present at the lumen, which form the microbiota. Thus the intestinal mucosa has to serve a complex barrier function, selectively allowing or denying the influx of luminal contents. Intestinal barrier function is built around a central structure, the epithelium, which constitutes the main obstacle to gaining access to the mucosa and that is in charge of regulating the selective transport of water, ions and nutrients. The other components of IBF include the mucus layer, immunoglobin A (IgA), antimicrobial peptides, and the mucosal immune system. Even the microbiota may be viewed as part of IBF, inasmuch as it is one important modulatory factor involved in its regulation. All these elements work in a highly integrated and interdependent manner. For instance, the microbiota influences epithelial dynamics (*i.e.*, proliferation and healing ability) both directly and indirectly [17]. Similarly, Toll-like receptor (TLR) 2 responds to microbiota derived peptidoglycan to enhance epithelial healing but it also stimulates trefoil factor (TFF) 3 production, which is a protective peptide that is part of the mucus layer [18].

Maintenance of IBF appears to be of paramount importance for the host. Evidence obtained in animal models as well as in humans is accumulating in support of a role of alterations of IBF in a vast array of conditions, which include intestinal disorders such as inflammatory bowel disease and irritable bowel syndrome but also obesity and metabolic syndrome, hepatic fibrosis and inflammation, sepsis, pancreatitis, and many others [17]. This in turn opens up the possibility of managing these diseases by reinforcing IBF, especially in terms of prevention. In this regard, the use of dietary products, such as diet-derived biopeptides in the management of chronic diseases is attractive because of the possibility of avoiding at least partly the side effects frequently associated with pharmacotherapy. Thus the investigative effort to develop new functional foods that are capable of providing benefit in these patients has increased notably in the last few years.

Different bioactive peptides have been proposed to treat hepatitis and colitis, in which alteration in IBF are considered to play a role. Animal models of colitis and ileitis have been used to study the effects of bovine glycomacropeptide [19,20,21,22,23], of an enzymatic hydrolysate of corn gluten [24], of pyroglutamyl leucine (a bioactive peptide from wheat gluten hydrolysate) [25] and of a β-casein hydrolysate generated by the cell envelope-associated proteinase of *Lactobacillus delbrueckii* ssp. CRL 581 [26]. Three of these studies used the trinitrobenzenesulfonic acid (TNBS) model of colitis. This is based on the administration to rat or mice of a single intrarectal dose of TNBS dissolved in ethanol. TNBS acts as a hapten that elicits an immune response when bound to tissue proteins, while ethanol contributes to the disruption of the intestinal barrier so that TNBS gains access to the mucosal milieu. The result is inflammation of the colon that shares several clinical and molecular features with Crohn’s disease [27]. The β-casein and the corn gluten hydrolysates were administered starting 10 days before the induction of colitis and they ameliorated inflammation in both cases. The β-casein hydrolysate additionally reduced microbial translocation to the liver, indicating a better intestinal mucosal barrier function [26]. Bovine glycomacropeptide has been shown to be anti-inflammatory not only in this animal model [19] but also in another model of chemically induced colitis, namely dextran sulfate sodium (DSS) induced colitis [28], as well as in the lymphocyte transfer model of colitis [23], and additionally in TNBS ileitis [21]. Because this is a glycosylated peptide it has been proposed that a prebiotic effect could be involved [19], although this hypothesis has not been formally tested. Modulation of the immune response has been also observed and in this regard glycomacropeptide has been shown to increase macrophage activity, to favor Treg differentiation and to hamper Th1 cell activation (see below) [20,21,29,30]. Last, it is worth noting that hepatic anti-inflammatory effects have been described also for the gluten derived peptide pyroglutamyl leucine [31].

The intestine of newborns has to deal with the introduction of the microbiota and with its special immature conditions that confer IBF a critical role at this developmental stage for the proper maturation of the newborn. Fermented milk-based infant formulas contain both dead bacteria and the products resulting from the fermentation process [32]. This type of formulas has been shown to be beneficial at the intestinal level and for infants IBF. A large study with 971 infants (four to six months of age) fed a fermented infant formula showed a decrease in the severity of diarrhea episodes when compared to standard formula fed infants [33]. No differences in incidence or duration of diarrhea episodes were observed in this study. Because secretory IgA (sIgA) is quite easy to measure, this parameter has been studied and found to be modified by the administration of fermented infant formulas. Higher poliovirus-specific IgA titres in response to Pentacoq vaccination were found in infants fed a fermented infant formula, from birth to 4 months of age, compared to those fed a non-fermented one [34]. This was a small study in which 30 infants were enrolled and only 20 completed the study. In another randomized and double blind study, pre-term infants fed a fermented infant formula showed higher fecal sIgA levels, but only when they had been partially breast fed, suggesting that fermented formula can boost the maternal IgA response [35].

## 4. Mucus

Mucus is the outermost protective layer of the intestinal mucosa. It consists of a gel overlying the epithelium and is based on the production and secretion of glycoproteins called mucins, mostly by goblet cells but also by regular enterocytes. Mucins bear substantial *O*-glycosylation and are either secreted (MUC2, 5AC, 5B and 6) by goblet cells or are membrane-associated (MUC1, 3, 4, 13 and 17), expressed by both goblet and absorptive cells in their apical membrane [36]. The mucus layer allows the passage of solutes while preventing luminal microorganisms from getting access to the epithelium. In normal conditions bacteria are found in the outer part of the mucus layer, where mucins may be degraded to some extent, but they for the most part do not progress much further. Thus the inner part of the layer is largely devoid of bacteria [36,37]. However, in pathologic conditions such as intestinal inflammation bacteria are more regularly found in contact with enterocytes, indicating a lack of containment by the mucus layer [37]. Reciprocally, absence of the main mucin, MUC2, results in spontaneous colitis and reduced antimicrobial defense in mice [38].

Casein enzymatic hydrolysates are known to stimulate rat intestinal mucus secretion, an effect that depends on opioid receptor stimulation [39] and that is exerted by bioactive peptides since neither the intact protein nor free amino acids have any reaction at this level. Among bovine β-casein derived bioactive peptides, β-casomorphins are a family that has shown μ-opioid receptor agonist activity. This family consists of β-casomorphin-4, -5, -6, and -7, which are obtained by cleavage of the 60–66 fragment of bovine β-casein [40]. Bovine-casomorphin-7 is the best studied and appears to be also the most efficient in mucus regulation [41]. Several studies have shown that it induces mucus secretion and mucin expression in goblet cells. Luminal and intra-arterial β-casomorphin-7 perfusion of rat jejunum results in enhanced mucus secretion, an effect that is inhibited by naloxone (a specific opiate receptor antagonist), indicating the requirement of peptide absorption and engagement of opioid receptors [41]. Induction of secretion has also been observed *in vitro* using human intestinal mucin producing cells (DHE and HT29/MTX), in which casomorphin-7 also enhanced the expression of mucin 2 and 3 in DHE cells, and additionally of mucin 5AC in HT29/MTX cells [42]. Another study has shown that other milk-derived peptides of either human or bovine origin also induce mucus secretion, with bovine β-lactorphin (β-lactoglobulin f(102–105)) being more potent than bovine β-casomorphin-7, which in turn is as potent as β-casomorphin-5 (β-casein f(51–55)) [43].

Mucus production and secretion can be also induced after oral administration of fermented milk products and peptides derived thereof [44,45,46]. Feeding mice for two days with the non-bacterial fraction of milk fermentation products of *Lactobacillus helveticus* (*L. helveticus*) and for three days with yoghurt or *L. casei* DN114 001 fermented milk supplemented diets increased the number of small intestine mucosal goblet cells [45]. Similarly, in a recent study, a total peptide fraction from yoghurt was shown to induce the secretion and production of mucins in HT29/MTX cells. Characterization of the responsible peptides led to the identification of β-casein f(94–123). Subsequent administration of this peptide to rat pups from postnatal day 10 to postnatal day 18 led to an increase in the number of goblet cells along the small intestine, and induced Muc2 and Muc4 expression in the small intestine [44]. In this study a hyperplasic effect on Paneth cells and an increased expression of antibacterial factors (lysozyme and Rdefa5) was also reported. A hyperplasic effect of bioactive peptides was also found after the dietary supplementation of mice with yoghurt or *L. casei* DN114 001 fermented milk [46].

### Immunoglobin A (IgA)

Type A immunoglobulins are a specialized form of antibodies found particularly in mucosal sites, although they are also present in serum and elsewhere. IgA occurs mainly in the form of dimers bound by an immunoglobulin J chain and featuring also the so called secretory component, which allows the epithelial cells to take up the dimers at the basolateral membrane and transport them for apical secretion. This form is called sIgA and is the predominant form of IgA. IgA is also secreted as monomers. One important feature of sIgA is that it is protected from proteolytic degradation, so that it is particularly well suited for luminal secretion as it endures the hostile environment in the gastrontestinal tract and other mucosal sites. Luminal sIgA is able to bind and block microbial antigens, thereby interfering with mechanisms of mucosal penetration and/or helping the immune system to fight off invading microbes once they are within reach. It also induces bacterial agglutination. Growing evidence indicates that IgA uses a high-affinity binding system to neutralize microbial toxins and pathogens, and a low-affinity binding system to prevent commensal bacteria from breaching the mucosal surface [47].

Reducing the production of IgA has important consequences for the host. For instance sIgA deficient mice show an increase in segmented filamentous bacteria [48] and in humans it has been associated with the development of inflammatory bowel disease-like inflammation [49]. It is important to note however that absence of IgA is at least partly compensated by adaptive measures such as an increased production of IgG and IgM antibodies [50].

Besides there are aforementioned studies in newborn children fed fermented infant formulas in which a stimulant effect on IgA production is described [34,35]. Studies in healthy animals consistently indicate that bioactive peptides from egg, milk, plants and fish proteins, as well as products that feature these peptides, potentiate IgA secretion [9,45,51,52,53,54,55,56]. In these studies IgA secretion is in general accompanied by an increase in the number of IgA^+^ cells in the small intestine and colonic mucosa. Furthermore, an increase in transforming growth factor (TGF)-β, interleukin-10 (IL-10) and IL-6, cytokines that induce an isotype switch in B cells from IgM to IgA [57], has been also described [9,45,52,53,56]. Animal models using pathogenic bacteria reinforce the evidence indicating that bioactive peptides enhance IgA secretion. Thus mice fed for 5 or 7 days the peptidic fraction of a *Lactobacillus helveticus* fermented milk were infected with *Escherichia coli* O157:H7 and showed more IgA^+^ B cells and higher intestinal and serum IgA after the infection than the control [58]. Similar results have been reported by Vinderola *et al.* in mice infected with *Salmonella enteriditis* serovar Typhimurium and fed with the non-bacterial fraction of milk fermented by *Lactobacillus helveticus* R389 [59]. Finally, a very interesting recent study focused not on milk derived peptides, but on a shark-derived protein hydrolysate, found an increase in the number of IgA producing cells in the intestine of healthy mice and in mice infected with enterotoxigenic *E. coli*, with concomitant up-regulation of the production of IL-6, tumor necrosis factor (TNF) α, TGF-β and IL-10 [52].

## 5. Intestinal Epithelial Cells

Intestinal epithelial cells are the most abundant cells of the intestinal epithelium and, beside their digestive and absorptive functions, they contribute to IBF in variety of ways including the maintenance of a physical monolayer barrier using tight junctions, the sensing of intestinal bacteria, the secretion of antimicrobial peptides and their overall contribution to the local immune response. The intestinal mucosa maintains a state of so-called “physiological inflammation”, *i.e.*, a low level activation of immune cells with infiltration of the lamina propria but devoid of clinical symptoms. Intestinal epithelial cells have been shown to contribute to this phenomenon through the production of cytokines as the result of their interaction with bacteria through TLRs. These receptors recognize not only microbial components, including proteins, lipids, and nucleic acids derived from bacteria, viruses and parasites, but also damaged host cell components such as nucleic acids and other “internal” ligands [60].

In the above-mentioned study with a shark-derived protein hydrolysate, the effect on IL-6 production by intestinal epithelial cells was assessed. Interestingly, in an *ex vivo* assay with primary cultures isolated form naive mice IL-6 production was increased and the induction depended on the stimulation of TLR2 and TLR4 [52]. Regulation of TLR-mediated signaling has been also described when colostrum isolated peptides were added to murine intestinal epithelial cells mIC_c12_ transfected with the luciferase gene under the control of NF-κB, the main transcription factor involved in TLR signaling [61]. While colostrum was found to reduce TLR-mediated signaling, peptides present in a colostrum undigested permeate (molecular weight (*M*_W_) < 3.5 kDa) stimulated NF-κB in basal conditions. The addition of this product to cells stimulated with peptidoglycan or Pam_3_CSK_4_, which are TLR2/NOD2 and TLR2/TLR1 agonists respectively, showed specific effects increasing peptidoglycan induced signal and inhibiting the signal evoked by Pam_3_CSK_4_. Interestingly the enzymatic digestion of the permeate abrogated its effect, indicating the peptidic nature of responsible substance. An attempt to identify the possible bioactive peptides indicated the presence of peptides originated from casein, pointing out the presence of endogenous enzymes in colostrum.

Intestinal epithelial cells are continuously renovated, and proliferation is key in the recovery of the epithelium after disruption by different insults that lead to intestinal inflammation. Therefore intestinal epithelial cell proliferation rate is very important to maintain IBF. In a study carried out with different fermented milk supernatants, yoghurt (obtained with *Streptococcus thermophilus* and *Lactobacillus bulgaricus* strain DN 540078) and *Lactobacillus paracasei* ssp. paracasei were shown to induce IEC6 cells proliferation and growth, yoghurt being more effective [62]. Bioactive peptides from bovine colostrum have also been shown to increase the proliferation of intestinal epithelial cells *in vitro*. *In vivo* digests of first day bovine colostrum obtained from calves increased the proliferation of human epithelial T84 cells [63]. In the same study *in vitro* digestion with pepsin and chymosin did not alter cell proliferation while digestion with trypsin inhibited it, suggesting that proteins of peptides inducing cell proliferation may be cleaved and therefore destroyed by trypsin. Colostrum has thus been proposed as a candidate source of bioactive peptides with potential aplication for gastrointestinal repair.

## 6. Mucosal Immune System

The intestinal mucosa is provided with an important branch of the immune system, which has the difficult task of protecting the intestinal tract while maintaining a non-inflammatory status despite the presence of massive amounts of bacteria and other microbes. The mucosal immune system of the intestine features various immune cell types such as neutrophils, monocyte/macrophages, dendritic cells, mast cells, innate lymphoid cells, B and T cells. As we have pointed above, epithelial cells are increasingly viewed as an integral part of the system. In addition to its direct role in microbe handling, the mucosal immune system is involved in other ways in intestinal homeostasis, since it contributes to IgA production and in the regulation of mucus generation and epithelial dynamics, as well as having an influence on the composition of the microbiota [17]. It is interesting that while idiopathic inflammation is not caused by any particular pathogenic microorganism, the occurrence of colitis (particularly in animals) requires the presence of luminal bacteria, indicating that the inflammatory response is directed largely toward the microbiota. On the other hand, total absence of the microbiota in germ free animals results in atrophy of the mucosal immune system with reduced numbers of cells and inflammatory markers and a diminished epithelial turnover. This has been related to higher susceptibility to noxious stimuli such as DSS. In turn, the presence of a normal microbiota is associated with a so-called “physiological inflammation”, a seemingly contradictory yet expressive expression that indicates the response of the immune system (compared with germ free conditions) unaccompanied by an inflammatory reaction [64]. This is accomplished by different adaptations of the mucosal immune system that globally lead to tolerance to the microbiota.

Reciprocally, a defect in the immune system may ultimately result in inflammation by weakening of IBF. For instance, inflammatory bowel disease-like inflammation has been described in a number of disorders associated with impaired lymphocyte function in humans [49]. Mice deficient in multiple components of the mucosal immune system are almost invariably prone to colitis or even develop spontaneous inflammation, including several TLRs and cytokines. Absence of neutrophils in rodents has also been related to increased susceptibility to colitis, although conflicting results have been reported. In line with the tolerance environment dominating the mucosal immune system, intestinal mucosal macrophages are unique in that they do not normally elicit inflammatory responses while maintaining intact their phagocytic and microbial killing capacity [65]. In addition, mucosal macrophages and dendritic cells are involved in tissue repair and immune tolerance in the gut. Monocytes are considered to contribute to inflammation nonetheless. As with neutrophils, conflicting results have been obtained with experiments of monocyte (and dendritic cell) depletion [66,67,68,69]. In humans, Crohn’s disease has been related to reduced/altered macrophage cytokine and bacterial clearance responses [70,71]. Interestingly, disorders of phagocyte function tend to result in Crohn’s disease-like chronic colitis [72,73].

Mainly two types of studies have been used to characterize the effects of bioactive peptides on immunity *in vivo*. In the first, one bioactive peptides are administered to healthy animals, resulting generally in immune-enhancing effects that potentiate IBF. Thus immune potentiating effects have been reported in healthy Balb/c mice fed an egg yolk low lipid peptic digest [55] or the non-bacterial fraction of milk fermented by *Lactobacillus helveticus* R389 [74]. The egg yolk hydrolysate increased the number of IL-10, IL-4, IL-12 and interferon (IFN) γ expressing cells and the phagocytic activity of murine peritoneal macrophages [55], while the number of IL-10, IL-2 and IL-6 positive cells and the secretion of IL-6 were increased in the small intestine of the animals fed the fermented milk [74]. A fish protein hydrolysate and a yellow field pea seed hydrolysate have also been tested with similar results (increase cytokine production of small intestine lamina propria cytokine and increased phagocytic activity) [53,56]. It is important to point out that these immune stimulant effects of bioactive peptides in healthy animals are not accompanied by any pathological features in any case.

In the second type mice or rats are given the product orally for days or weeks and then a bacterial toxin or toxic bacteria are administered in order to study the immune reaction, and inhibition is predominantly seen in this case. When the mice that received the shark hydrolysate were challenged with an experimental enterotoxigenic *E. coli* H10407, which induces diarrhea, they showed higher TGF-β serum levels and lower IL-17 levels in intestinal fluids [52]. Other pertinent studies include those performed with Balb/c mice fed the non-bacterial fraction of *L. helveticus* and challenged with either *Salmonella enteriditis* [59] or *E. coli* O157:H7 [58], both of which showed an improved mucosal immune response with an increased production of IL-4. Furthermore, and additional down-regulation of IFN-γ response in mice challenged with *E. coli* was observed [58].

An anti-inflammatory effect has also been described for QEPVL, a casein derived peptide that results from *L. helveticus* milk fermentation. The administration of this peptide to old male Balb/c mice for three weeks inhibited the immune response to injected lipopolissacharide (LPS). The results showed increased levels of circulating of anti-inflammatory cytokines IL-10 and IL-4 and an inhibition of inflammation associated TNFα and IFNγ [75]. Additional *in vitro* studies indicated that QEPVL and also QEPV increase proliferation and cAMP levels in mouse primary lymphocytes [75]. cAMP has shown anti-inflammatory effects on immune regulation by increasing the expression of IL-10, among other mechanisms.

Spleen cell preparations (frequently referred to as splenocytes) contain several cell types including macrophages, dendritic cells, B cells and T lymphocytes, and are commonly used for immunomodulatory studies *in vitro*. Because cytokines released by cells affect the behavior of other cell types, the use of splenocytes allows the observation of the overall effect in a mixed immune cell population. Splenocytes have been used to characterize the effect of bioactive peptides such as bovine glycomacropeptide [20] and an enzymatic hydrolysate from the algae *Porphyra columbina* [76]. A hydrolysate from *Porphyra columbina*, obtained by digestion with flavourizyme and a fungal protease concentrate, showed an increase in splenocyte proliferation in basal and concanavalin A (ConA) stimulated conditions and of IL-10 expression in basal, lipopolysaccharide (LPS) and ConA stimulated conditions. LPS evokes cytokine production in TLR4 expressing cells and, among splenocytes, macrophages are considered the main cytokine producers after LPS stimulation. In turn, ConA is a plant mitogen, which is able to stimulate T cells. Therefore these results may be globally viewed as indicative of anti-inflammatory effects on lymphocytes and macrophages. This hypothesis was further studied in isolated macrophages and T lymphocytes isolated from rat spleen. The hydrolysate increased the proliferation of both cell types and induced an anti-inflammatory cytokine profile inhibiting the expression of TNFα and IL-6 in macrophages while enhancing IL-10 production in both cell types. An increase in IFNγ was observed after exposure of ConA stimulated T cells to the hydrolysate, suggesting a pivotal role of macrophages in the overall response. Mechanistically, the hydrolysate induced IL-10 by c-Jun *N*-terminal kinase (JNK), p38 mitogen-activated protein kinase (MAPK) and NF-κB dependent pathways in T lymphocytes [76].

We have described that bovine glycomacropeptide inhibits the production of IFNγ in rat splenocytes and increases their proliferation when stimulated with ConA [20]. It also induces cyclooxigenase 2, inducible nitric oxide synthase, IL-10 and FoxP3 expression. These results may be interpreted to indicate enhancement of macrophage activity and Treg differentiation, and inhibition of Th1 cell activation. It should be noted that conflicting results have been obtained in this regard, with inhibited splenocyte proliferation in the presence of both LPS and phytohemagglutinin (a T cell mitogen) being observed in one study [77]. Nevertheless, and in accordance with our results, in another study carried out with the human macrophague cell line U937, bovine glycomacropeptide potentiated proliferation and phagocytic activity [30]. In addition, in THP-1 cells (another monocyte/macrophague cell line) and in human peripheral blood macrophages this peptide enhanced pro-inflammatory cytokine production (IL-1β, TNFα and IL-8) [29]. In this last study, the characterization of signal transduction pathways involved the activation of MAPKs p38, JNK and extracellular signal-regulated kinases (ERK) and particularly of the NF-κB signal transduction pathways.

It should be noted that even though *in vitro* studies with isolated macrophages show in general immunostimulatory effects of bioactive peptides when added to cells in the basal state, anti-inflammatory actions are regularly found in stimulated macrophages. When a cell free supernatant obtained from milk fermented by *L. helveticus* was added to RAW264.7 cells (a murine macrophage cell line), an enhanced phagocytic activity together with an increase in cytokine (TNFα, IL-6 and IL-1β) and nitric oxide production was observed. Two of nine fractions collected from the supernatant using size exclusion chromatography produced the highest response when used to stimulate macrophages. Characterization of peptides in one of these fractions led to the identification of 4 β-caseins (f(145–160), f(145–154), f(143–154) and f(192–202)) and one α-lactalbumin (f(115–122)) peptide as possible candidates for this effect [78]. These observations are in agreement with the increased activity of peritoneal macrophages described in mice orally administered a *L. helveticus* fermented milk [51].

Soybean, amaranth, yellow field pea seeds, common bean (*Phaseolus vulgaris* L.) and almond derived peptides reportedly down-regulate pro-inflammatory cytokine production in activated macrophages and modulate their phagocytic activity. Thus, a higher macrophage phagocytic activity was observed when an alcalase soy protein hydrolysate was added to peritoneal macrophages [79]. In addition, soybean proteins digested with alcalase inhibit inflammatory markers (inducible nitric oxide synthase (iNOS), prostaglandin (PG) E_2_, cyclooxigenase (COX)-2) in the macrophage cell line RAW 264.7 activated with LPS [80,81]. This cell line was also used to describe the anti-inflammatory effect of commercially available soymilk products digested sequentially with pepsin and pancreatin [82]. A decreased nitric oxide and IL-1β production together with an inhibition in the expression of NOS and COX-2 was described in LPS stimulated macrophages. Similarly, yellow field pea seed and almond hydrolysates significantly inhibited NO production and the secretion of pro-inflammatory cytokines in activated RAW264.7 cells [56,83]. A 5 kDa fraction isolated from the almond hydrolysate retained this effect, modulating the production of IL-6, IL-1β, TNFα, iNOS and COX-2 in stimulated cells [83]. Industrial processes can modify the anti-inflammatory properties of hydrolysates. In this regard, extrusion has been shown to increase the anti-inflammatory effect of amaranthus hydrolysates in LPS stimulated macrophage cell lines (THP1 and RAW264.7). These studies show that both extruded and unprocessed pepsin/pancreatin hydrolysates were anti-inflammatory, but the extrusion hydrolysate was more potent [84,85]. Prevention of NF-κB activation, as demonstrated by a reduced phosphorylation of IκB-α, was described as the mechanism of action. The inhibition of the NF-κB signal transduction pathway by bioactive peptides in macrophages also has been described as the mechanism of action of an alcalase hydrolysate of common bean and of lunasin, a soybean-derived peptide with antioxidant and anticarcinogenic properties [86,87,88]. In fact, lunasin has been shown to down-regulate LPS induced pro-inflammatory signaling in THP-1 interacting with αVβ3 integrin receptor and inhibiting the activation of phosphorylated Akt [86] and NF-κB [86,88].

The T cell response to hydrolysates has been assessed *in vitro*. Treg cells are one of the main producers of IL-10, an anti-inflammatory protein that induces the down-regulation of Th1 cells and the enhancement of B cell survival, proliferation and antibody production, among other actions. As commented above for bovine glycomacropeptide and *Porphyra columbina* hydrolysates, bioactive peptides seem to stimulate proliferation, to increase the expression of IL-10 in isolated T cells, and to inhibit the Th1 response[20,29,76]. These effects have been shown for other products. For instance, while β-lactoglobulin acidic tryptic-chymotryptic peptides stimulate splenocyte proliferation and IFN-γ production, *in vitro* hydrolysis of these peptides with Lactobacillus paracasei NCC2461 peptidases repressed lymphocyte stimulation, up-regulated IL-10 production, and down-regulated IFN-γ and IL-4 secretion [89]. Besides, studies with a yak milk hydrolysate indicate a different modulation of T cells. These peptides increase IFN-γ and IL-2, which are key cytokines for Th1 cell development, in a dose-dependent manner, with no obvious effects on IL-4 secretion, which is more closely related to Th2 cells [90].

## 7. Conclusions

There is compelling evidence supporting the biological relevance of peptides released by either natural or artificial means from a number of dietary sources. These appear to act at different levels of the intestinal barrier, and the overall effect is consistent with reinforcement of IBF and protection of the host. However, the characterization of the biopeptides responsible for such actions is clearly insufficient. Given the variety expected from peptides generated from different sources and digestion processes, it is surprising that their actions appear to be very much alike (Figure 1) In order to gain acceptance as products useful in the management of IBF related diseases it is important to go deeper into the specific peptides involved in these effects and the mechanisms involved.

## References

[1] Korhonen H. (2009). Milk-derived bioactive peptides: From science to applications. J. Funct. Foods.

[2] Fan X., Bai L., Zhu L., Yang L., Zhang X. (2014). Marine algae-derived bioactive peptides for human nutrition and health. J. Agric. Food Chem..

[3] Chakrabarti S., Jahandideh F., Wu J. (2014). Food-derived bioactive peptides on inflammation and oxidative stress. Biomed. Res. Int..

[4] Beermann C., Hartung J. (2013). Physiological properties of milk ingredients released by fermentation. Food Funct..

[5] Yu Z., Yin Y., Zhao W., Chen F., Liu J. (2014). Application and bioactive properties of proteins and peptides derived from hen eggs: Opportunities and challenges. J. Sci. Food Agric..

[6] Ryan J.T., Ross R.P., Bolton D., Fitzgerald G.F., Stanton C. (2011). Bioactive peptides from muscle sources: Meat and fish. Nutrients.

[7] Nasri R., Nasri M. (2013). Marine-derived bioactive peptides as new anticoagulant agents: A review. Curr. Protein Pept. Sci..

[8] Kim J.A., Kim S.K. (2013). Bioactive peptides from marine sources as potential anti-inflammatory therapeutics. Curr. Protein Pept. Sci..

[9] Vinderola G., Perdigon G., Duarte J., Farnworth E., Matar C. (2006). Effects of the oral administration of the products derived from milk fermentation by kefir microflora on immune stimulation. J. Dairy Res..

[10] Chaves-Lopez C., Serio A., Paparella A., Martuscelli M., Corsetti A., Tofalo R., Suzzi G. (2014). Impact of microbial cultures on proteolysis and release of bioactive peptides in fermented milk. Food Microbiol..

[11] Zagato E., Mileti E., Massimiliano L., Fasano F., Budelli A., Penna G., Rescigno M. (2014). *Lactobacillus paracasei* CBA L74 metabolic products and fermented milk for infant formula have anti-inflammatory activity on dendritic cells *in vitro* and protective effects against colitis and an enteric pathogen *in vivo*. PLoS One.

[12] Galdeano C.M., Nunez I.N., de LeBlanc A.D., Carmuega E., Weill R., Perdigon G. (2011). Impact of a probiotic fermented milk in the gut ecosystem and in the systemic immunity using a non-severe protein-energy-malnutrition model in mice. BMC Gastroenterol..

[13] Nunez I.N., Galdeano C.M., Carmuega E., Weill R., de LeBlanc A.D., Perdigon G. (2013). Effect of a probiotic fermented milk on the thymus in Balb/c mice under non-severe protein-energy malnutrition. Br. J. Nutr..

[14] Klaenhammer T.R., Kleerebezem M., Kopp M.V., Rescigno M. (2012). The impact of probiotics and prebiotics on the immune system. Nat. Rev. Immunol..

[15] Besselink M.G.H., van Santvoort H.C., Buskens E., Boermeester M.A., van Goor H., Timmerman H.M., Nieuwenhuijs V.B., Bollen T.L., van Ramshorst B., Witteman B.J.M. (2008). Probiotic prophylaxis in predicted severe acute pancreatitis: A randomised, double-blind, placebo-controlled trial. Lancet.

[16] Tsilingiri K., Barbosa T., Penna G., Caprioli F., Sonzogni A., Viale G., Rescigno M. (2012). Probiotic and postbiotic activity in health and disease: Comparison on a novel polarised *ex vivo* organ culture model. Gut.

[17] Sanchez de Medina F., Romero-Calvo I., Mascaraque C., Martinez-Augustin O. (2014). Intestinal inflammation and mucosal barrier function. Inflamm. Bowel Dis..

[18] Podolsky D.K., Gerken G., Eyking A., Cario E. (2009). Colitis-associated variant of TLR2 causes impaired mucosal repair because of TFF3 deficiency. Gastroenterology.

[19] Daddaoua A., Puerta V., Zarzuelo A., Suarez M.D., Sanchez de Medina F., Martinez-Augustin O. (2005). Bovine glycomacropeptide is anti-inflammatory in rats with hapten-induced colitis. J. Nutr..

[20] Requena P., Gonzalez R., Lopez-Posadas R., Abadia-Molina A., Suarez M.D., Zarzuelo A., de Medina F.S., Martinez-Augustin O. (2010). The intestinal antiinflammatory agent glycomacropeptide has immunomodulatory actions on rat splenocytes. Biochem. Pharmacol..

[21] Requena P., Daddaoua A., Martínez-Plata E., González M., Zarzuelo A., Suárez M.D., Sánchez de Medina F., Martínez-Augustin O. (2008). Bovine glycomacropeptide ameliorates experimental rat ileitis by mechanisms involving down-regulation of interleukin 17. Br. J. Pharmacol..

[22] De Medina F.S., Daddaoua A., Requena P., Capitan-Canadas F., Zarzuelo A., Suarez M.D., Martinez-Augustin O. (2010). Session 9: Food ingredients, immunity and inflammation: Animal and *in vitro* models new insights into the immunological effects of food bioactive peptides in animal models of intestinal inflammation. Proc. Nutr. Soc..

[23] Ortega-Gonzalez M., Capitan-Canadas F., Requena P., Ocon B., Romero-Calvo I., Aranda C., Suarez M.D., Zarzuelo A., Sanchez de Medina F., Martinez-Augustin O. (2014). Validation of bovine glycomacropeptide as an intestinal anti-inflammatory nutraceutical in the lymphocyte-transfer model of colitis. Br. J. Nutr..

[24] Mochizuki M., Shigemura H., Hasegawa N. (2010). Anti-inflammatory effect of enzymatic hydrolysate of corn gluten in an experimental model of colitis. J. Pharm. Pharmacol..

[25] Wada S., Sato K., Ohta R., Wada E., Bou Y., Fujiwara M., Kiyono T., Park E.Y., Aoi W., Takagi T. (2013). Ingestion of low dose pyroglutamyl leucine improves dextran sulfate sodium-induced colitis and intestinal microbiota in mice. J. Agric. Food Chem..

[26] Turbay M.B.E., de LeBlanc A.D., Perdigon G., de Giori G.S., Hebert E.M. (2012). β-Casein hydrolysate generated by the cell envelope-associated proteinase of *Lactobacillus delbrueckii* ssp. lactis CRL 581 protects against trinitrobenzene sulfonic acid-induced colitis in mice. J. Dairy Sci..

[27] Martinez-Augustin O., Merlos M., Zarzuelo A., Suarez M.D., Sanchez de Medina F. (2008). Disturbances in metabolic, transport and structural genes in experimental colonic inflammation in the rat: A longitudinal genomic analysis. BMC Genomics.

[28] Lopez-Posadas R., Requena P., Gonzalez R., Suarez M.D., Zarzuelo A., Sanchez de Medina F., Martinez-Augustin O. (2010). Bovine glycomacropeptide has intestinal antiinflammatory effects in rats with dextran sulfate-induced colitis. J. Nutr..

[29] Requena P., Daddaoua A., Guadix E., Zarzuelo A., Suarez M.D., Sanchez de Medina F., Martinez-Augustin O. (2009). Bovine glycomacropeptide induces cytokine production in human monocytes through the stimulation of the MAPK and the NF-κB signal transduction pathways. Br. J. Pharmacol..

[30] Li E.W., Mine Y. (2004). Immunoenhancing effects of bovine glycomacropeptide and its derivatives on the proliferative response and phagocytic activities of human macrophagelike cells, U937. J. Agric. Food Chem..

[31] Sato K., Egashira Y., Ono S., Mochizuki S., Shimmura Y., Suzuki Y., Nagata M., Hashimoto K., Kiyono T., Park E.Y. (2013). Identification of a hepatoprotective peptide in wheat gluten hydrolysate against d-galactosamine-induced acute hepatitis in rats. J. Agric. Food Chem..

[32] Granier A., Goulet O., Hoarau C. (2013). Fermentation products: Immunological effects on human and animal models. Pediatr. Res..

[33] Thibault H., Aubert-Jacquin C., Goulet O. (2004). Effects of long-term consumption of a fermented infant formula (with *Bifidobacterium breve* c50 and *Streptococcus thermophilus* 065) on acute diarrhea in healthy infants. J. Pediatr. Gastroenterol. Nutr..

[34] Mullie C., Yazourh A., Thibault H., Odou M.F., Singer E., Kalach N., Kremp O., Romond M.B. (2004). Increased poliovirus-specific intestinal antibody response coincides with promotion of *Bifidobacterium longum-infantis* and *Bifidobacterium breve* in infants: A randomized, double-blind, placebo-controlled trial. Pediatr. Res..

[35] Campeotto F., Suau A., Kapel N., Magne F., Viallon V., Ferraris L., Waligora-Dupriet A.J., Soulaines P., Leroux B., Kalach N. (2011). A fermented formula in pre-term infants: Clinical tolerance, gut microbiota, down-regulation of faecal calprotectin and up-regulation of faecal secretory IgA. Br. J. Nutr..

[36] Kim Y.S., Ho S.B. (2010). Intestinal goblet cells and mucins in health and disease: Recent insights and progress. Curr. Gastroenterol. Rep..

[37] Johansson M.E., Gustafsson J.K., Holmen-Larsson J., Jabbar K.S., Xia L., Xu H., Ghishan F.K., Carvalho F.A., Gewirtz A.T., Sjovall H. (2014). Bacteria penetrate the normally impenetrable inner colon mucus layer in both murine colitis models and patients with ulcerative colitis. Gut.

[38] Sharpe S.M., Qin X.F., Lu Q., Feketeova E., Palange D.C., Dong W., Sheth S.U., Lee M.A., Reino D., Xu D.Z. (2010). Loss of the intestinal mucus layer in the normal rat causes gut injury but not toxic mesenteric lymph nor lung injury. Shock.

[39] Claustre J., Toumi F., Trompette A., Jourdan G., Guignard H., Chayvialle J.A., Plaisancie P. (2002). Effects of peptides derived from dietary proteins on mucus secretion in rat jejunum. Am. J. Physiol..

[40] Teschemacher H., Koch G., Brantl V. (1997). Milk protein-derived opioid receptor ligands. Biopolymers.

[41] Trompette A., Claustre J., Caillon F., Jourdan G., Chayvialle J.A., Plaisancie P. (2003). Milk bioactive peptides and β-casomorphins induce mucus release in rat jejunum. J. Nutr..

[42] Zoghbi S., Trompette A., Claustre J., El Homsi M., Garzon J., Scoazec J.Y., Plaisancie P. (2006). β-Casomorphin-7 regulates the secretion and expression of gastrointestinal mucins through a μ-opioid pathway. Am. J. Physiol..

[43] Martinez-Maqueda D., Miralles B., de Pascual-Teresa S., Reveron I., Munoz R., Recio I. (2012). Food-derived peptides stimulate mucin secretion and gene expression in intestinal cells. J. Agric. Food Chem..

[44] Plaisancie P., Claustre J., Estienne M., Henry G., Boutrou R., Paquet A., Leonil J. (2013). A novel bioactive peptide from yoghurts modulates expression of the gel-forming MUC2 mucin as well as population of goblet cells and Paneth cells along the small intestine. J. Nutr. Biochem..

[45] Vinderola G., Matar C., Perdigon G. (2007). Milk fermentation products of *L. helveticus* R389 activate calcineurin as a signal to promote gut mucosal immunity. BMC Immunol..

[46] Thoreux K., Balas D., Bouley C., Senegas-Balas F. (1998). Diet supplemented with yoghurt or milk fermented by *Lactobacillus casei* DN-114 001 stimulates growth and brush-border enzyme activities in mouse small intestine. Digestion.

[47] Macpherson A.J., Mccoy K.D., Johansen F.E., Brandtzaeg P. (2008). The immune geography of IgA induction and function. Mucosal Immunol..

[48] Suzuki K., Meek B., Doi Y., Muramatsu M., Chiba T., Honjo T., Fagarasan S. (2004). Aberrant expansion of segmented filamentous bacteria in IgA-deficient gut. Proc. Natl. Acad. Sci. USA.

[49] Marks D.J., Seymour C.R., Sewell G.W., Rahman F.Z., Smith A.M., McCartney S.A., Bloom S.L. (2010). Inflammatory bowel diseases in patients with adaptive and complement immunodeficiency disorders. Inflamm. Bowel Dis..

[50] Slack E., Hapfelmeier S., Stecher B., Velykoredko Y., Stoel M., Lawson M.A., Geuking M.B., Beutler B., Tedder T.F., Hardt W.D. (2009). Innate and adaptive immunity cooperate flexibly to maintain host-microbiota mutualism. Science.

[51] Matar C., Valdez J.C., Medina M., Rachid M., Perdigon G. (2001). Immunomodulating effects of milks fermented by *Lactobacillus helveticus* and its non-proteolytic variant. J. Dairy Res..

[52] Mallet J.F., Duarte J., Vinderola G., Anguenot R., Beaulieu M., Matar C. (2014). The immunopotentiating effects of shark-derived protein hydrolysate. Nutrition.

[53] Duarte J., Vinderola G., Ritz B., Perdigon G., Matar C. (2006). Immunomodulating capacity of commercial fish protein hydrolysate for diet supplementation. Immunobiology.

[54] LeBlanc J.G., Matar C., Valdez J.C., LeBlanc J., Perdigon G. (2002). Immunomodulating effects of peptidic fractions issued from milk fermented with *Lactobacillus helveticus*. J. Dairy Sci..

[55] Nelson R., Katayama S., Mine Y., Duarte J., Matar C. (2007). Immunomodulating effects of egg yolk low lipid peptic digests in a murine model. Food Agric. Immunol..

[56] Ndiaye F., Vuong T., Duarte J., Aluko R.E., Matar C. (2012). Anti-oxidant, anti-inflammatory and immunomodulating properties of an enzymatic protein hydrolysate from yellow field pea seeds. Eur. J. Nutr..

[57] Cerutti A. (2008). The regulation of IgA class switching. Nat. Rev. Immunol..

[58] LeBlanc J., Fliss I., Matar C. (2004). Induction of a humoral immune response following an *Escherichia coli* O157:H7 infection with an immunomodulatory peptidic fraction derived from *Lactobacillus helveticus*-fermented milk. Clin. Vaccine Immunol..

[59] Vinderola G., Matar C., Perdigon G. (2007). Milk fermented by *Lactobacillus helveticus* R389 and its non-bacterial fraction confer enhanced protection against *Salmonella enteritidis* serovar Typhimurium infection in mice. Immunobiology.

[60] Sanchez de Medina F., Ortega-Gonzalez M., Gonzalez-Perez R., Capitan-Canadas F., Martinez-Augustin O. (2013). Host-microbe interactions: The difficult yet peaceful coexistence of the microbiota and the intestinal mucosa. Br. J. Nutr..

[61] Malinowski J., Klempt M., Clawin-Radecker I., Lorenzen P.C., Meisel H. (2014). Identification of a NF-κB inhibitory peptide from tryptic β-casein hydrolysate. Food Chem..

[62] Thoreux K., Senegas-Balas F., Bernard-Perrone F., Giannarelli S., Denariaz G., Bouley C., Balas D. (1996). Modulation of proliferation, second messenger levels, and morphotype expression of the rat intestinal epithelial cell line IEC-6 by fermented milk. J. Dairy Sci..

[63] Morgan A.J., Riley L.G., Sheehy P.A., Wynn P.C. (2014). The influence of protein fractions from bovine colostrum digested *in vivo* and *in vitro* on human intestinal epithelial cell proliferation. J. Dairy Res..

[64] Hooper L.V., Littman D.R., Macpherson A.J. (2012). Interactions between the microbiota and the immune system. Science.

[65] Cario E. (2010). Toll-like receptors in inflammatory bowel diseases: A decade later. Inflamm. Bowel Dis..

[66] Berndt B.E., Zhang M., Chen G.H., Huffnagle G., Lai K., Zhang J., Kao J.Y. (2007). The role of dendritic cells in the development of acute dextran sulfate sodium colitis. Gastroenterology.

[67] Steinbach E.C., Plevy S.E. (2014). The role of macrophages and dendritic cells in the initiation of inflammation in IBD. Inflamm. Bowel Dis..

[68] Qualls J.E., Kaplan A.M., van Rooijen N., Cohen D.A. (2006). Suppression of experimental colitis by intestinal mononuclear phagocytes. J. Leukoc. Biol..

[69] Qualls J.E., Tuna H., Kaplan A.M., Cohen D.A. (2009). Suppression of experimental colitis in mice by CD11c^+^ dendritic cells. Inflamm. Bowel Dis..

[70] Marks D.J.B., Harbord M.W.N., MacAllister R., Rahman F.Z., Young J., Al-Lazikani B., Lees W., Novelli M., Bloom S., Segal A.W. (2006). Defective acute inflammation in Crohn’s disease: A clinical investigation. Lancet.

[71] Smith A.M., Rahman F.Z., Hayee B., Graham S.J., Marks D.J.B., Sewell G.W., Palmer C.D., Wilde J., Foxwell B.M.J., Gloger I.S. (2009). Disordered macrophage cytokine secretion underlies impaired acute inflammation and bacterial clearance in Crohn’s disease. J. Exp. Med..

[72] Rahman F.Z., Marks D.J., Hayee B.H., Smith A.M., Bloom S.L., Segal A.W. (2008). Phagocyte dysfunction and inflammatory bowel disease. Inflamm. Bowel Dis..

[73] Marks D.J., Miyagi K., Rahman F.Z., Novelli M., Bloom S.L., Segal A.W. (2009). Inflammatory bowel disease in CGD reproduces the clinicopathological features of Crohn’s disease. Am. J. Gastroenterol..

[74] Vinderola G., Matar C., Palacios J., Perdigon G. (2007). Mucosal immunomodulation by the non-bacterial fraction of milk fermented by *Lactobacillus helveticus* R389. Int. J. Food Microbiol..

[75] Jiehui Z., Liuliu M., Haihong X., Yang G., Yingkai J., Lun Z., Li D.X., Dongsheng Z., Shaohui Z. (2014). Immunomodulating effects of casein-derived peptides QEPVL and QEPV on lymphocytes *in vitro* and *in vivo*. Food Funct..

[76] Cian R.E., Lopez-Posadas R., Drago S.R., Sanchez de Medina F., Martinez-Augustin O. (2012). A Porphyra columbina hydrolysate up-regulates IL-10 production in rat macrophages and lymphocytes through an NF-κB, and p38 and JNK dependent mechanism. Food Chem..

[77] Otani H., Monnai M., Kawasaki Y., Kawakami H., Tanimoto M. (1995). Inhibition of mitogen-induced proliferative responses of lymphocytes by bovine κ-caseinoglycopeptides having different carbohydrate chains. J. Dairy Res..

[78] Tellez A., Corredig M., Brovko L.Y., Griffiths M.W. (2010). Characterization of immune-active peptides obtained from milk fermented by *Lactobacillus helveticus*. J. Dairy Res..

[79] Kong X., Guo M., Hua Y., Cao D., Zhang C. (2008). Enzymatic preparation of immunomodulating hydrolysates from soy proteins. Bioresour. Technol..

[80] Vernaza M.G., Dia V.P., de Mejia E.G., Chang Y.K. (2012). Antioxidant and anti-inflammatory properties of germinated and hydrolysed Brazilian soybean flours. Food Chem..

[81] Martinez-Villaluenga C., Dia V.P., Berhow M., Bringe N.A., Gonzalez de Mejia E. (2009). Protein hydrolysates from β-conglycinin enriched soybean genotypes inhibit lipid accumulation and inflammation *in vitro*. Mol. Nutr. Food Res..

[82] Dia V.P., Bringe N.A., de Mejia E.G. (2014). Peptides in pepsin-pancreatin hydrolysates from commercially available soy products that inhibit lipopolysaccharide-induced inflammation in macrophages. Food Chem..

[83] Udenigwe C.C., Je J.Y., Cho Y.S., Yada R.Y. (2013). Almond protein hydrolysate fraction modulates the expression of proinflammatory cytokines and enzymes in activated macrophages. Food Funct..

[84] Montoya-Rodriguez A., de Mejia E.G., Dia V.P., Reyes-Moreno C., Milan-Carrillo J. (2014). Extrusion improved the anti-inflammatory effect of amaranth (*Amaranthus hypochondriacus*) hydrolysates in LPS-induced human THP-1 macrophage-like and mouse RAW 264.7 macrophages by preventing activation of NF-κB signaling. Mol. Nutr. Food Res..

[85] Montoya-Rodriguez A., Milan-Carrillo J., Dia V.P., Reyes-Moreno C., Gonzalez de Mejia E. (2014). Pepsin-pancreatin protein hydrolysates from extruded amaranth inhibit markers of atherosclerosis in LPS-induced THP-1 macrophages-like human cells by reducing expression of proteins in LOX-1 signaling pathway. Proteome Sci..

[86] Cam A., de Mejia E.G. (2012). RGD-peptide lunasin inhibits Akt-mediated NF-κB activation in human macrophages through interaction with the αVβ3 integrin. Mol. Nutr. Food Res..

[87] Oseguera-Toledo M.E., de Mejia E.G., Dia V.P., Amaya-Llano S.L. (2011). Common bean (*Phaseolus vulgaris* L.) hydrolysates inhibit inflammation in LPS-induced macrophages through suppression of NF-κB pathways. Food Chem..

[88] De Mejia E.G., Dia V.P. (2009). Lunasin and lunasin-like peptides inhibit inflammation through suppression of NF-κB pathway in the macrophage. Peptides.

[89] Prioult G., Pecquet S., Fliss I. (2004). Stimulation of interleukin-10 production by acidic β-lactoglobulin-derived peptides hydrolyzed with *Lactobacillus paracasei* NCC2461 peptidases. Clin. Diagn. Lab. Immun..

[90] Mao X.Y., Yang H.Y., Song J.P., Li Y.H., Ren F.Z. (2007). Effect of yak milk casein hydrolysate on TH1/TH2 cytokines production by murine spleen lymphocytes *in vitro*. J. Agric. Food Chem..

